# St. Jude's Offertories

**Published:** 1917-01-20

**Authors:** William C. Parsons


*To the Editor of* The Hospital.

### St. Jude's Offertories.

*To the Editor of* The Hospital.

Sir,—The amount deducted for church expenses from St. Jude's offertories on December 31 was £3 from a total of £13 14s., the Red Cross Society and the Belgian Relief Fund receiving £5 7s. each. I am afraid I cannot realise our criminality in our endeavour to be "just before we are generous." Our church expenses have a way of running on whether the object is national or otherwise, and I cannot see any reason for letting the Red Cross Society and Hospital Sunday Fund free from contributing their quota towards the upkeep of the Church.

I cannot follow your remarks that when the congregation fail to provide the means to maintain its efficiency there must be something wrong with those responsible for the services and the church's management. The fact remains that certain expenses have to be met or the church must be closed.—I am, Sir, yours faithfully,

William C. Parsons.

[Why the Belgian Relief Fund on Red Cross Sunday? Every church worthy of continuance in this country should be managed on sound principles of business. Then a congregation will always support the House of God, where they attend for worship and prayer. An absence of spiritual life and the substitution of convention and a dull routine properly produces impoverishment—*i.e.*, a failure to provide the means. This demonstrates the "something wrong." Without life and system a Church must fail.—Ed. The Hospital.]

